# Soy Isoflavone Genistein Impedes Cancer Stemness and Mesenchymal Transition in Head and Neck Cancer through Activating miR-34a/RTCB Axis

**DOI:** 10.3390/nu12071924

**Published:** 2020-06-29

**Authors:** Pei-Ling Hsieh, Yi-Wen Liao, Chang-Wei Hsieh, Pei-Ni Chen, Cheng-Chia Yu

**Affiliations:** 1 Department of Anatomy, School of Medicine, China Medical University, Taichung 40402, Taiwan; plhsieh@mail.cmu.edu.tw; 2 School of Dentistry, Chung Shan Medical University, Taichung 40201, Taiwan; rabbity0225@gmail.com; 3 Department of Medical Research, Chung Shan Medical University Hospital, Taichung 40201, Taiwan; 4 Department of Food Science and Biotechnology, National Chung Hsing University, Taichung 402, Taiwan; welson@nchu.edu.tw; 5 Institute of Biochemistry, Microbiology and Immunology, Chung Shan Medical University, Taichung 40201, Taiwan; peini@csmu.edu.tw; 6 Institute of Oral Sciences, Chung Shan Medical University, Taichung 40201, Taiwan; 7 Department of Dentistry, Chung Shan Medical University Hospital, Taichung 40201, Taiwan

**Keywords:** cancer stemness, genistein, head and neck cancer, Microrna-34a, RTCB

## Abstract

Genistein, a soy-derived phytoestrogen, has been shown to exhibit anti-neoplastic activities in various cancers. Nevertheless, its effects on the elimination of tumor-initiating cells of head and neck cancer (HNC-TICs) remain unclear. Here, we investigated the inhibitory effect of genistein on HNC-TICs and potential mechanisms. Our results demonstrated that genistein lowered the proliferation of HNC-TICs by examining the percentage of ALDH1+ or CD44+ cells. Aside from the downregulation of epithelial-mesenchymal transition (EMT) in HNC-TICs, genistein restricted their tumor propagating capacities in a dose-dependent fashion. Moreover, genistein potentiated cell death caused by three commonly used chemotherapeutic agents (doxorubicin, cisplatin, and 5-FU). Our findings proved that genistein induced ROS production through upregulation of miR-34a, leading to apoptosis in HNC-TICs. The genistein-elicited miR-34a reduced self-renewal, migration, invasion capacities and ALDH1 activity, which may be partly owing to the repression of EMT. Furthermore, we showed that RTCB was a novel target that was negatively regulated by miR-34a and involved in the tumor repressive effect of genistein. Besides, the in vivo study validated that genistein retarded tumor growth through the elevation of miR-34a and suppression of RTCB. These results suggested that genistein-induced miR-34a contributed to the ROS-associated apoptosis and diminished stemness properties via repression of RTCB in HNC-TICs.

## 1. Introduction

Head and neck cancer (HNC) includes various malignancies that originate in the squamous cell mucosa or lining of the head and neck regions. It has been shown that the incidence of HNC increased over the past three decades [1] and HNC still constitutes around 5% of new cases of cancer in 2018 [2]. Aside from the loco-regional spread, a significant number of patients (~13.8%) developed distant metastases and had dismal prognosis since the median time from distant metastasis to death was only 3.3 months [3]. It has been revealed that tumor-initiating cells (TICs)/cancer stem cells (CSCs), a small subgroup of cells within a malignant clonal population that possess the ability to self-renew and differentiate, are associated with metastasis and tumor recurrence. Hence, these findings highlighted the importance of preventing metastasis and an urgent need to explore therapeutic agents to eradicate TICs. In an attempt to develop approaches to target TICs, a variety of methods have been used to identify and enrich these cells, such as sphere-formation [4], ALDH1 [5], and CD44 staining [6]. On the other hand, it has been suggested that reactive oxygen species (ROS) may activate various transcription factors that are implicated in cellular transformation, tumorigenesis, and metastasis [7]. Paradoxically, accumulating evidence has suggested that ROS modulate the expression of numerous tumor suppressors as well [8], and may eliminate cancer cells by selectively inducing apoptosis [9]. Previously, it has been revealed that there was a subset of HNC cells exhibited a lower level of intracellular ROS and displayed TICs properties with enhanced malignant potential and chemoresistance [10]. Moreover, Chang et al. showed that the antioxidant capacity of TICs was critical to maintaining the stemness features [10], suggesting that an elevation of ROS in HNC-TICs may downregulate their stemness characteristics due to the oxidant-antioxidant imbalance and improve the efficacy of conventional chemotherapy. Given that TICs contribute to metastasis, drug resistance and tumor recurrence [11], developing approaches that elevate the concentration of ROS in TICs has become an emerging trend [12,13].

Genistein (4′,5,7-trihydroxyisoflavone) is a major constituent of *Genista tridentata* L. and a phytoestrogen belonging to the class of isoflavones, which can be found in various soybean foods [14]. It has been shown to have a number of benefits in human health, such as atheroprotective [15] and anti-cancer [16] effects. Genistein has been considered as a mitochondriotropic agent to modulate the mitochondrial redox biology [17], and was found to elicit cell cycle arrest, apoptosis and inhibit invasion in an HNC cell line HN4 cells [18,19,20]. Another study showed that genistein inhibited HNC cell line SCC-25 cell growth via G2/M phase arrest and was able to suppress cycloxygenase-2 activity [21]. As for in vivo study, it has been revealed that the blood vessel density and VEGF mRNA expression were significantly downregulated in the genistein-treated nude mice bearing HNC cell line HSC-3 cells [22]. Furthermore, genistein has been demonstrated to attenuate TICs features in several cancers, such as breast [23], prostate [24] and gastric [25,26] cancers. It has been shown that genistein inhibited the stemness properties of these TICs via Hedgehog-Gli1 pathway [23,24,25] or reduced chemoresistance through inhibition of ABCG2 expression and ERK 1/2 activity [26]. As to nasopharyngeal TICs, it also has been revealed that genistein suppressed cell proliferation and induced apoptosis via Sonic Hedgehog signaling [27]. Moreover, numerous studies have shown that genistein exerted the anti-tumor effects through the upregulation of miR-34a [28,29]. As a type of non-coding RNAs (RNA that does not encode a protein), microRNAs (~19–22 nucleotides) have been known to participate in the regulation of cancer stemness of oral cancer [30]. Various studies have revealed that miR-34a suppressed the characteristics of TICs and prevented metastasis in prostate cancer or breast cancer through repressing CD44 [31] or Notch1 [32], respectively. It appears that genistein possesses the anti-HNC capacity and may exert an inhibitory effect through regulation of miR-34a, but this hypothesis is not yet verified. Additionally, it was imperative to investigate whether genistein affects drug sensitivity and elucidate the detailed mechanism of its effect.

To this end, we isolated and enriched the patient-derived HNC-TICs and treated these cells with various concentrations of genistein followed by the analysis of TICs features to assess the anti-stemness properties of genistein. Apart from testing the effect of genistein on the sensitization of chemotherapy, we also assessed the ROS production and examined if the induced ROS resulted in apoptosis. Besides, we examined whether genistein these actions through modulation of miR-34a. Most importantly, we uncovered a novel downstream target of miR-34a, RTCB, which was a 3’-phosphate RNA ligase that has not been well-characterized. Altogether, we revealed the mechanism underlying the repressive activities of genistein in HNC-TICs.

## 2. Materials and Methods

### 2.1. Cell Culture and Chemical Compounds

This study was approved by the Institutional Review Board of China Medical University Hospital (CSMUH: No: CS13250). HNC tissues were resected from two HNC patients who gave informed consent for the use of their tissues that were harvested at the surgery. To identify and ALDH1+CD44+ HNC-TICs and ALDH1-CD44- non-TICc, we stained cancer cells with ALDEFLUOR™ assay kit (StemCell Technologies, Vancouver, BC, Canada) and anti-CD44 antibody conjugated to phycoerythrin (BioLegend, San Diego, CA, USA) followed by fluorescence-activated cell sorting using FACSAria II cell sorter (BD Biosciences, San Jose, CA, USA). Smulow–Glickman (S-G) human gingival epithelial cells were originally derived from human attached gingiva [33].

Genistein (G-6649; Sigma, St Louis, MO, USA) and anti-oxidant NAC (N-acetyl-l-cysteine) were purchased from Sigma Chemical Co. (St. Louis, MO, USA). Genistein was dissolved in DMSO as a stock solution and diluted in culture medium to final concentrations (10–80 μM) prior to use.

### 2.2. Cell Proliferation Assay

Cell proliferation/survival was evaluated by MTT assay. First, 1 × 10^4^ cells/well in DMSO or various concentrations of genistein-containing medium were added in a 96-well plate and cultured at 37 °C for 24 h followed by incubation with MTT reagent for 3 h. The blue formazan crystals were dissolved in DMSO and then measured at 570 nm using Infinite 200 PRO plate reader (Tecan, Männedorf, Switzerland). The effects of chemotherapies were examined by the MTT assay. We chose the concentration of genistein (<IC50) for further study.

### 2.3. Secondary Sphere Assay

Cells were dissociated and cultured in the serum-free DMEM/F12 medium supplemented with N2, human recombinant bFGF, EGF (R&D Systems, Minneapolis, MN, USA), and penicillin/streptomycin at 10^3^ cells/low-attachment 6-well plate (Corning Inc., Corning, NY, USA). The culture medium was changed every other day [4].

### 2.4. Flow Cytometry

ALDEFLUOR assay kit (StemCell Technologies, Durham, NC, USA) was used to examine the ALDH1 positive cells according to the manufacture protocol. As for CD44 expression, cells were stained with dilution 1:100 anti-CD44 antibody conjugated to phycoerythrin (Miltenyi Biotech., Auburn, CA, USA) and detected by flow cytometry (FACSCaliburTM, BD Biosciences, San Jose, CA, USA) using CellQuest software.

### 2.5. Migration and Invasion Assays

The migration and invasion abilities were evaluated using the 24-well Corning Transwell cell culture system with 8 μm pore size. The membrane was coated with Matrigel for invasion assay. Cell suspensions were seeded in the upper compartment (1 × 10^5^ cells/well) and 10% serum served as a chemoattractant in the lower chamber. After 24 h, the cells on the filter membrane facing the lower chamber were stained with crystal violet (Sigma-Aldrich). The migrated and invasion cells were then visualized by microscope at 100× and counted from 5 different visual areas.

### 2.6. Colony Formation Analysis

Colony formation units were determined by soft-agar assay. A bottom layer of agar mixture [DMEM, 10% (*v/v*) FCS, 0.6% (*w/v*) agar] was poured and solidified in a six-well culture dish followed by the addition of an upper layer containing 2 × 10^4^ cells suspended in agar-medium-mixture [DMEM, 10% (*v/v*) FCS, 0.3% (*w/v*) agar]. After 4 weeks, plates were stained with crystal violet and the colonies were counted.

### 2.7. Real-Time qRT-PCR

Total RNA was prepared from cells using Trizol reagent according to the manufacturer’s protocol (Invitrogen, Carlsbad, CA, USA) and reversely transcribed by Superscript III firsTt-strand synthesis system (Invitrogen). qRT-PCR on resulting cDNAs was performed on an ABI StepOnePlus™ Real-Time PCR System (Applied Biosystems, Life Technologies Corp., Carlsbad, CA, USA) by SYBR Green reagent with specific primers. Given the acquisition of metastatic ability has been considered to be associated with EMT, which promoted cell migration and invasion through upregulation of several E-cadherin suppressors, such as Snail, Slug, and ZEB1 (Pearson, London, UK, 2019). At the same time, the cytoskeletal intermediate filaments of these cells underwent a compositional change and initiated the expression of vimentin (Mendez et al., 2010 [34]). We chose to examine E-cadherin, Snail, Slug, ZEB1 and vimentin. GAPDH is used for internal control. The primer sequences are listed below (Table 1):

### 2.8. Western Blotting

The proteins of HNC-TICs were extracted using RIPA buffer. Samples were boiled and separated by 10% SDS-PAGE. The proteins were wet transferred to polyvinylidene difluoride membrane (Amersham, Arlington Heights, IL, USA). After blocking, the membranes were incubated with appropriate primary antibodies against Snail, ZEB1, vimentin, Slug, or E-cadherin followed by corresponding secondary antibodies. The immunoreactive bands were developed using the ECL-plus chemiluminescence substrate (Perkin-Elmer, Waltham, MA, USA) and detected by ImageQuant LAS 4000 Mini (GE Healthcare, Piscataway, NJ, USA). The detailed information of antibodies are listed below (Table 2).

### 2.9. Self-Renewal Assay

For self-renewal capacity, primary 1 × 10^4^ spheres were dissociated and cells were treated with genistein and various chemotherapeutic agents, or transfected with overexpression of miR-34a or RTCB and let to form spheres. After 1 or 2 weeks, secondary spheres were counted by microscope (DMi8, Leica, Wetzlar, Germany) and presented as percentage of the control group.

### 2.10. Overexpression of miR-34a and miR-34a Inhibitor

MiR-34a mimic, scramble (Scr) control, and miR-34a inhibitor were purchased from Applied Biosystems. HNC-TICs were transfected with LipofectamineTM 3000 transfection reagent (Invitrogen) following the manufacturer’s instructions. Scrambled oligos (Scr) were used as transfection control. Mature miR-34a Sequence: UGGCAGUGUCUUAGCUGGUUGU.

### 2.11. Assessment of Apoptosis

HNC-TICs (5 × 10^5^ cells) were treated with genistein alone or combined with NAC/miR34a inhibitor treatment for 24 h. The treated cells were collected and subjected to annexin V and PI staining by using Vybrant Apoptosis Assay Kit 2 (Invitrogen, Carlsbad, CA, USA) according to the manufacture’s protocol. The apoptotic cells were analyzed by FACSCaliburTM (BD BioSciences, San Jose, CA, USA).

### 2.12. ROS Analysis

The ROS production was assessed by flow cytometry as the fluorescence of 2′,7′-dichlorofluorescein (DCF) and ethidium (ETH), which are the oxidation products of 2′,7′- dichlorodihydrofluorescein diacetate (DCFH-DA; Sigma-Aldrich, Madrid, Spain) and dihydroethidium (DHE; Molecular Probes, OR, USA) with a sensitivity for H2O2/NO-based radicals and O-2, respectively. ETH fluorescence and DCF fluorescence of 10,000 cells was analyzed by flow cytometry at 488 nm and quantified the results by FlowJo software (TreeStar; Ashland, OR, USA).

### 2.13. Analysis of Luciferase Activity

The pmirGLO-RTCB-Wt reporter was generated by cloning wild-type putative target region of RTCB to pmirGLO plasmids (Promega, Madison, WI, USA) according to the manufacturer’s instructions. The pmirGLO-RTCB-mut reporter was generated using a site-directed mutagenesis kit (Clontech, San Francisco, CA, USA). Then, 1 × 10^4^ Cells grown in 96-well plates were co-transfected with 50 ng pmirGLO-RTCB-Wt reporter, 50 ng pmirGLO-RTCB-mut reporter, miR34a mimics, or miR-Scr using Lipofectamine 2000 reagent followed by the analysis of luciferase activity using Infinite 200 PRO plate reader (Tecan, Männedorf, Switzerland). Firefly luciferase activity normalized against renilla luciferase activity, which was used to represent transfection efficiency, is presented as reporter activity in this study.

### 2.14. Subcutaneous Xenografts in Nude Mice

All procedures involving animals were performed in accordance with the institutional animal welfare guidelines of the Institutional Animal Care and Use Committee (IACUC) at the Chung Shan Medical University (approval code: 1368). HNC-TICs (1 × 10^4^ cells/0.2 mL/mouse) were injected subcutaneously into BALB/c nude mice (6–8 weeks). Eight days postimplantation, the mice were randomly divided into three groups (*N* = 5 for each group) and fed by oral gavage with saline (control) or genistein (25 and 50 mg/day/kg) suspended in saline. Bioluminescence imaging was performed using an IVIS50 animal imaging system (Xenogen Corp., Alameda, CA, USA). The displayed images of the tumor sites were drawn and quantified in photons per second using Living Image software (Xenogen Corp.). The volume (cm^3^) was calculated according to the following formula: [length × width^2^]/2).

### 2.15. Statistical Analysis

SPSS (version 13.0; SPSS, Inc., Chicago, IL, USA) was used for statistical analysis. ANOVA analysis was used to determine the statistical significance of the differences among experimental groups. Pearson’s correlation coefficient was used to evaluate the correlation between miR-34a and RTCB. *p* values less than 0.05 were considered statistically significant. The presented results were representative of three independent experiments with similar results.

## 3. Results

### 3.1. Genistein Inhibits the Cell Growth of HNC-TIC

**Figure 1:**
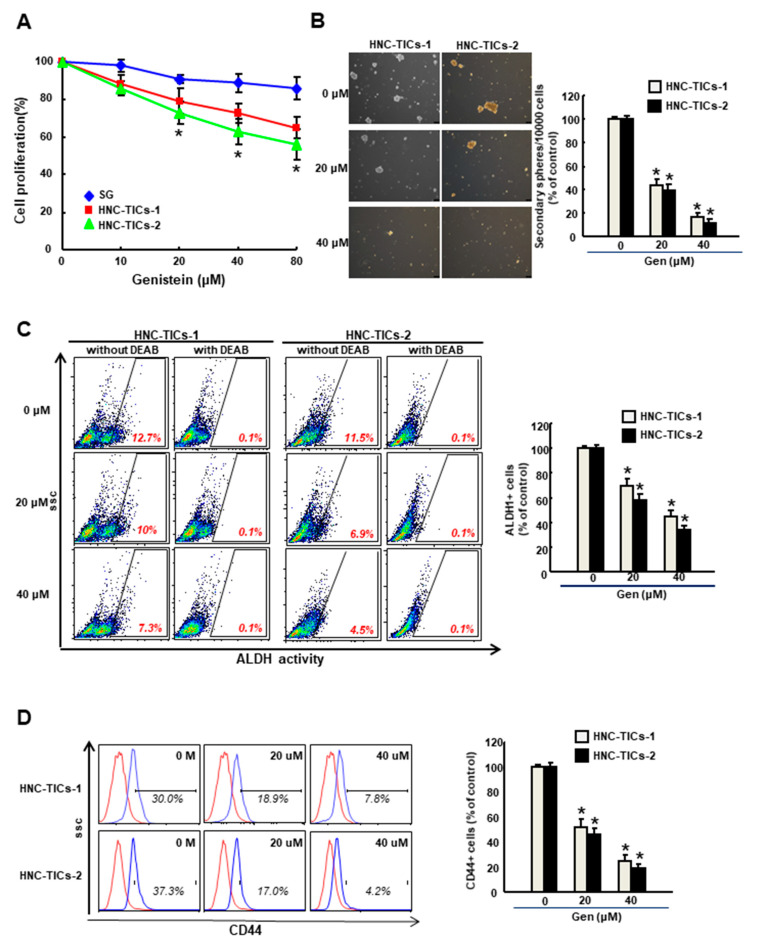
(**A**) Cell proliferation of normal human gingival epithelioid S-G (SG) cells and two lines of patient-derived HNC-TICs (tumor-initiating cells of head and neck cancers) in response to various concentrations of genistein (Gen) using MTT assay; (**B**) Percentage of HNC-TICs formed secondary spheres. Original magnification: 100×; scale bar: 100 μm; the proportion of (**C**) aldehyde dehydrogenase 1 (ALDH1)-expressing and (**D**) CD44-expressing cells in HNC-TICs following treatment of various concentration of genistein using flow cytometry. N,N-diethylaminobenzaldehyde (DEAB) was used as a selective inhibitor of ALDH1. * *p* < 0.05 compared to no treatment.

As shown in Figure 1A, the application of genistein dose-dependently diminished the cell proliferation of two lines of patient-derived HNC-TICs without damaging the normal S-G cells. The ability of the dissociated HNC-TIC to form second-generation spheres was reduced by genistein as well (Figure 1B). The decreased proportion of the ALDH1+ (Figure 1C) and CD44+ (Figure 1D) cells in response to genistein coincided with the observation of lower cell survival and self-renewal capacity after genistein treatment.

### 3.2. Genistein Suppresses the Stemness Phenotypes and Epithelial-Mesenchymal Transition (EMT) Traits of HNC-TIC

**Figure 2:**
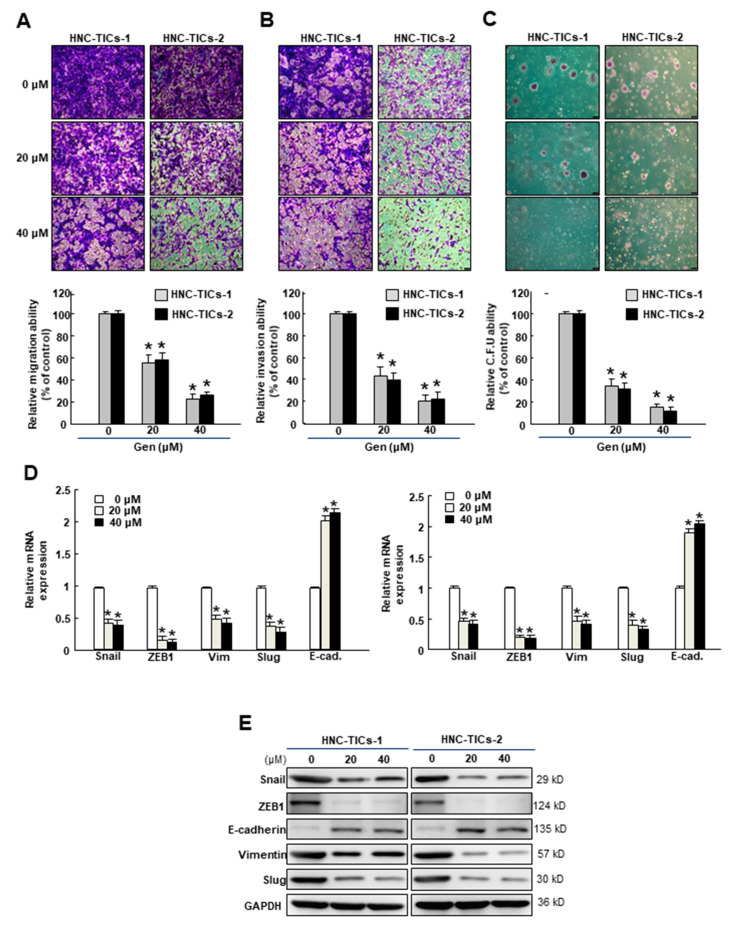
A number of stemness properties, including (**A**) migration, (**B**) invasion, and (**C**) colony-forming abilities of HNC-TICs (tumor-initiating cells of head and neck cancers) were examined using Transwell system or soft-agar assay; Original magnification: 100×; scale bar: 100 μm. The (**D**) gene and (**E**) protein expression of several EMT markers, including ZEB1 (zinc finger E-box binding homeobox 1), E-cad (E-cadherin), Vim (vimentin), and Slug were measured following 24-h administration of genistein (Gen) using qRT-PCR or Western blot. * *p* < 0.05 compared to no treatment.

Genistein exerted the repressive capacity to lessen various stemness characteristics, including migration (Figure 2A), invasion (Figure 2B) and colony-forming abilities (Figure 2C). Here, we demonstrated that genistein downregulated the expression levels of the EMT inducers, Snail, ZEB1, and Slug as well as vimentin. In addition, the expression of E-cadherin was upregulated (Figure 2D,E and Table 1). Taken together, we showed that genistein holds the potential for preventing pro-metastatic events via regulation of EMT markers.

### 3.3. Genistein Increases the Chemosensitivities and Downregulates the Stemness Features of HNC-TIC

**Figure 3:**
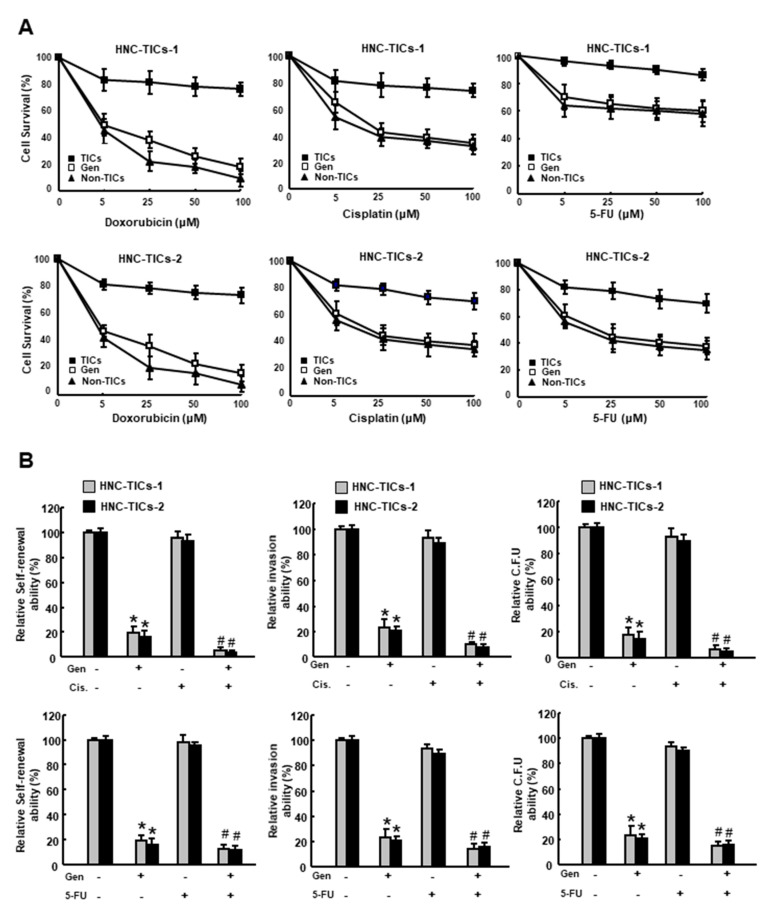
(**A**) Cell survival in response to multiple chemotherapies of non-TICs (non-tumor-initiating cells), TICs (tumor-initiating cells) and TICs treated with genistein (20 µM) using two HNC-TICs (tumor-initiating cells of head and neck cancers) was tested using MTT assay; (**B**) Self-renewal ability following treatment of genistein (Gen) with or without Cisplatin (Cis.)/ 5-Fluorouracil (5-FU). * *p* < 0.05 compared to control group. # *p* < 0.05 compared to genistein only group.

The ability of HNC-TIC to circumvent various drug regimens has been thought to be one of the greatest impediments to cancer therapy. As shown in Figure 3A, the cell survival of two HNC-TICs remained high in response to doxorubicin, cisplatin, or 5-fluorouracil (5-FU) treatments compared to Non-TICs. Nevertheless, co-administration of chemotherapies and genistein successfully enhanced chemosensitization of these three drugs, respectively. Moreover, the abilities of self-renewal, invasion and colony formation were improved in the genistein-treated cells (Figure 3B), suggesting that genistein may be suitable to serve as an adjunct to low dose chemotherapies and prevent unfavorable side effects induced by chemotherapies.

### 3.4. Genistein Induces Apoptosis of HNC-TIC via miR-34a-Mediated Oxidative Stress

**Figure 4:**
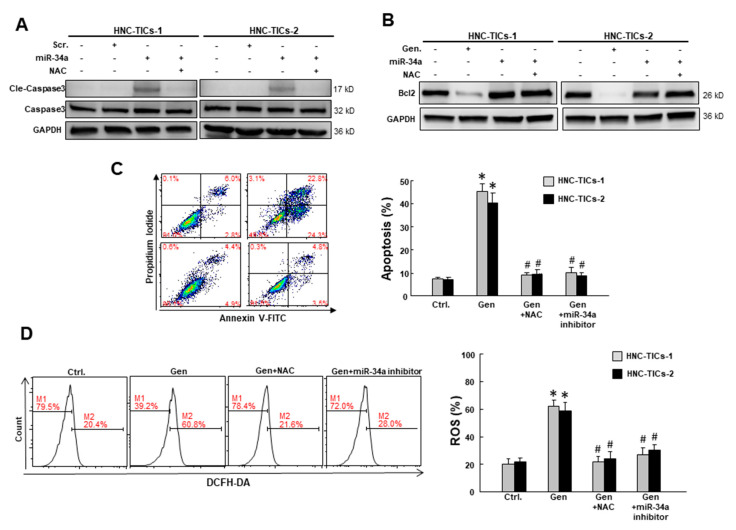
(**A**) The expression of the cleaved caspase-3 and caspase-3 in HNC-TICs (tumor-initiating cells of head and neck cancers) with scr. (scramble), overexpression of miR-34a or co-treatment with NAC (N-acetylcysteine) using Western blot; (**B**) The expression of Bcl-2 in HNC-TICs with treatment of genistein (Gen) (20 µM) or combination of NAC (1 mM) or miR-34a inhibitor (1 μM) was analyzed by Western blotting; GAPDH (glyceraldehyde-3-phosphate dehydrogenase) was used as the internal control. (**C**) Percentage of apoptotic cells and (**D**) ROS in two HNC-TICs following administration of genistein (20 µM), genistein (20 µM)+ NAC (1 mM), and genistein (20 µM)+ miR-34a inhibitor (1 μM) were presented using flow cytometry. * *p* < 0.05 compared to control group. # *p* < 0.05 compared to genistein only group.

The upregulation of miR-34a by genistein has been shown to inhibit cell growth and induce apoptosis in pancreatic cancer cells (Xia et al., 2012 [28]). As a result, we overexpressed miR-34a and found that the expression of cleaved caspase-3 was upregulated, while co-treatment with anti-oxidant N-acetylcysteine (NAC) prevented the elevation of cleaved caspase-3 (Figure 4A). This finding suggested that the miR-34a-induced expression of cleaved caspase-3 required the elevation of oxidative stress. Subsequently, we demonstrated that genistein exhibited a similar capacity to downregulate expression of Bcl2 (Figure 4B). Likewise, NAC treatment or miR-34 inhibition impeded the genistein-induced apoptosis in HNC-TIC (Figure 4B). The result from flow cytometry was consistent with the protein expression of apoptosis markers and showed that the percentage of apoptotic cells increased in the genistein-treated cells (Figure 4C). However, this upregulation was reversed by NAC or miR-34a inhibitor (Figure 4C), indicating that the ability of genistein to elicit apoptosis in HNC-TIC may be through miR-34a-mediated oxidative stress. To verify this hypothesis, we conducted the DCFH-DA assay and showed that ROS production was indeed elevated in the genistein-treated cells, but this upregulation was not observed in cells treated with NAC or miR-34a inhibitor (Figure 4D).

### 3.5. The Repressed Stemness Phenotypes and EMT Traits by Genistein Is through Activation of miR-34a

**Figure 5:**
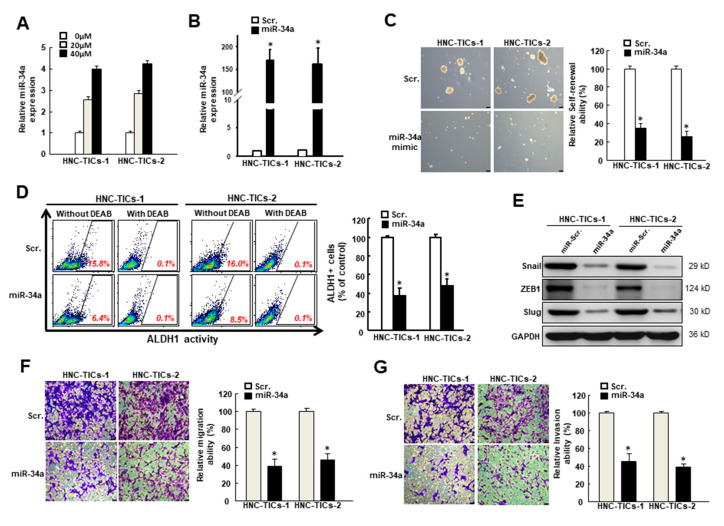
(**A**) The relative expression of miR-34a in two HNC-TICs (tumor-initiating cells of head and neck cancers) after treatment of various concentration of genistein using real-time qRT-PCR; (**B**) The transfection efficiency of scramble (Scr) and miR-34a mimic-transfected cells was measured using real-time qRT-PCR; (**C**) Relative self-renewal ability and (**D**) ALDH1 activity of two HNC-TICs with scramble control or overexpression of miR-34a; (**E**) The expression of Snail, ZEB1 (zinc finger E-box binding homeobox 1), and Slug in HNC-TICs with overexpression of miR-34a using Western blotting; GAPDH (glyceraldehyde-3-phosphate dehydrogenase) was used as the internal control. Relative (**F**) migration and (**G**) invasion capacities in miR-34a-overexpressing TICs. Original magnification: 100×; scale bar: 100 μm * *p* < 0.05 compared to scramble control.

We then sought to investigate whether miR-34a was implicated in the suppressive effect of genistein on HNC-TIC. As expected, the expression of miR-34a was dose-dependently increased as the concentration of genistein increased (Figure 5A). Next, we assessed the effect of overexpressed miR-34a (Figure 5B) and showed that the self-renewal capacity (Figure 5C), ALDH1 activity (Figure 5D), the expression of EMT inducers (Figure 5E), migration (Figure 5F) and invasion (Figure 5G) abilities were all downregulated by ectopic expression of miR-34a. These results suggested that the upregulation of miR-34a displayed anti-cancer properties and may diminish the progression of EMT.

### 3.6. The miR-34a-Depressed Stemness Properties Are via Downregulation of RTCB

**Figure 6:**
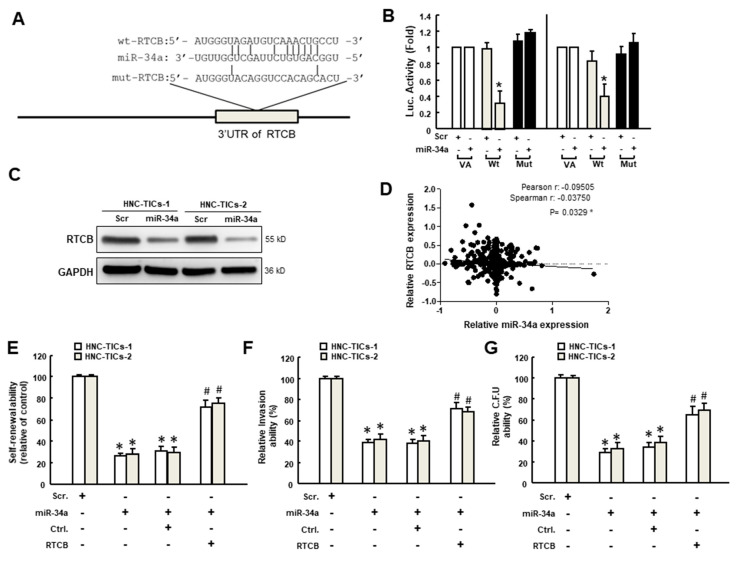
(**A**) Schematic presentation of the constructed 3′ untranslated region (UTR) reporter plasmids of RTCB (RNA 2′,3′-cyclic phosphate and 5′-OH ligase). Wild-type (Wt) and mutated (Mut) RTCB reporter plasmids were co-transfected with miR-34a or empty vectors (vector alone; VA); (**B**) The luciferase activity of each combination in two HNC-TICs (tumor-initiating cells of head and neck cancers) was assessed and only WT reporter activity was suppressed by miR-34a; (**C**) The expression of RTCB in HNC-TICs with scramble or miR-34a overexpression; GAPDH (glyceraldehyde-3-phosphate dehydrogenase) was used as the internal control. (**D**) The inverse correlation between RTCB and miR-34a in HNC samples using The Cancer Genome Atlas (TCGA) dataset; (**E**) Self-renewal (**F**), invasion or (**G**) colony-forming abilities in miR-34a-overexpressing cells with or without RTCB overexpression were evaluated. * *p* < 0.05 compared to scramble control. # *p* < 0.05 compared to miR-34a overexpression group.

To unravel the target gene of miR-34a that participated in the anti-cancer effects, we used bioinformatics software (Target Scan program) and predicted that RTCB may be a potential target. RTCB is an RNA ligase that catalyzed unconventional RNA splicing during unfolded protein response (UPR) (Lu et al., 2014 [35]), which has been regarded to be associated with metastasis and chemoresistance (Madden et al., 2019 [36]). In order to confirm the direct relationship between miR-34a and RTCB, we constructed the reporter plasmids containing either full-length (wild-type) or mutated forms of the 3′-untranslated region (3′UTR) region of RTCB (Figure 6A) as microRNAs have been known to post-transcriptionally regulate the translational efficiency or stability of targeted mRNAs by directly hybridizing to the 3′-UTR of their targets. In both HNC-TICs, the luciferase activity of reporter plasmids containing full-length RTCB 3′UTR was downregulated, while the activity was not affected in the mutated form of RTCB (Figure 6B). Besides, the protein expression of RTCB was inhibited in the miR-34a-overexpressing cells (Figure 6C and Table 2) and we observed there was a negative correlation between miR-34a and RTCB in HNC tissues from The Cancer Genome Atlas (TCGA) database (Figure 6D). Furthermore, the self-renewal ability, invasion capacity and colony-forming property were all repressed in miR-34a-overexpressing cells, whereas the forced expression of RTCB reversed these phenomena. Altogether, these results demonstrated that the reduced cancer stemness characteristic by miR-34a was through the suppression of RTCB.

### 3.7. Genistein Attenuates the Oncogenicity in Vivo through Upregulation of miR-34a

**Figure 7:**
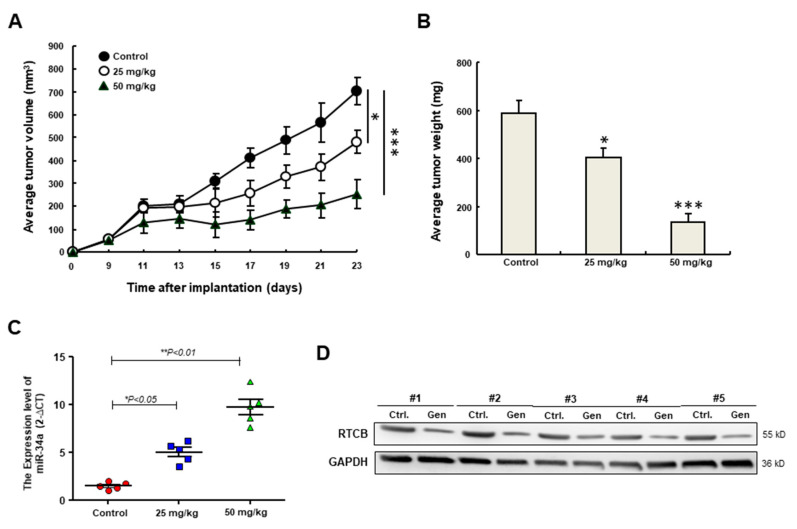
(**A**) Tumor volume and (**B**) tumor weight of subcutaneous HNC-TICs xenografts in nude mice treated with the indicated concentration of genistein (Gen); (**C**) The relative gene expression of miR-34a and (**D**) the protein expression of RTCB (RNA 2′,3′-cyclic phosphate and 5′-OH ligase) in the excised tumors. GAPDH (glyceraldehyde-3-phosphate dehydrogenase) was used as the internal control. * p < 0.05; *** p < 0.01.

Given that the repressive effect of genistein on oncogenic features and EMT traits has been demonstrated in vitro, it was crucial to carry out the in vivo validation. As shown in Figure 7A,B, genistein remarkably reduced the tumor volume and tumor weight in a dose-dependent manner. In agreement with the in vitro data, the expression of miR-34a was dose-dependently increased following genistein treatment in the excised tumor tissues (Figure 7C). Furthermore, the expression of RTCB was consistently reduced in the 50 mg/kg genistein-treated group (Figure 7D). Collectively, these results showed that genistein exerted anti-TIC properties through the miR-34a/RTCB axis.

## 4. Discussion 

In the present study, we showed that genistein was able to downregulate the cell growth and reduce the aggressiveness of HNC-TICs, which was consistent with previous studies showing genistein was capable of inhibiting HNC cells [18,20] and attenuating TICs features in other cancers [23,26]. Suppression of the HNC-TICs proliferation by genistein also resulted in the overall reduction in drug resistance as TICs have been known to express high levels of ATP-binding cassette (ABC) transporters which contribute to chemoresistance. Our results demonstrated that the effect of these commonly used chemotherapy drugs on cell survival of non-TICs was evident. Nevertheless, the considerable chemoresistance of TICs restricted their effectiveness. With a combination of genistein, the cell viability of TICs remarkably reduced. Besides, the self-renewal, invasion, and colony-forming capacities were attenuated as well. These results indicated that genistein exerted a synergistic effect with chemotherapeutic agents on the inhibition of TICs compared with either agent alone. One of the recent reviews has indicated that genistein and its metabolites interact with ABC transporters, which mediate multidrug resistance in cancer cells [37]. Moreover, it has been unraveled that there were numerous binding sites for EMT-inducing transcription factors in the promoters of ABC transporters, and overexpression of these factors, such as Snail, did increase the promoter activity of ABC transporters [38]. Since genistein was able to modulate the expression of EMT inducers and inhibit ABC transporters, further studies are required to elucidate the exact mechanism underlying the effect of genistein for sensitization of HNC-TICs to chemotherapies.

Extensive evidence suggested that microRNAs participated in a variety of cellular events in cancer cells, including the maintenance of stemness [30]. As a member of the miR-34 family, miR-34a has long been known to be directly transactivated by p53, resulting in induced apoptosis and cell-cycle arrest [39,40]. Various anti-apoptotic proteins, such as Bcl2, was found to be direct targets of miR-34a [41]. In agreement with this finding, we showed that the expression of Bcl2 was downregulated following genistein treatment, which may be due to the miR-34a repression. Furthermore, we found that both the administration of genistein and ectopic miR-34a expression induced the upregulation of cleaved caspase-3, possibly owning to the reduced inhibition from Bcl2 [42]. Moreover, the expression of Bcl2 was reversed by genistein+ antioxidant NAC, indicating that ROS was necessary for the genistein-inhibition of Bcl2. In conjunction with the finding that miR-34a-mediated caspase-3 cleavage was associated with the production of ROS, it was likely that ROS contributed to the inhibition of Bcl2 by miR-34a. Furthermore, the genistein-induced ROS was downregulated by miR-34a inhibitor, suggesting that the generation of ROS was due to miR-34a upregulation. This hypothesis was supported by a previous study showing that miR-34a mimics increased chemotherapy drug-induced ROS production in retinoblastoma cells [43]. Collectively, our results indicated that excessive ROS as a result of genistein-induced miR-34a may render Bcl2 more likely to be targeted by miR-34a, leading to caspase-3 activation and apoptosis.

In addition to apoptosis, we demonstrated that forced expression of miR-34a diminished various oncogenic features, which was consistent with previous findings that showed miR-34a significantly downregulated in HNC tumors and cell lines [44]. Meanwhile, we demonstrated that numerous EMT inducers, including Snail, ZEB1 and Slug were repressed by overexpression of miR-34a, which was in conformity with a study showing that the upregulation of miR-34a caused suppression of EMT inducers [45]. Siemens et al. showed that miR-34a directly regulated Snail expression through binding to the 3′UTR of Snail. Aside from downregulation of Slug and ZEB1, miR-34a also suppressed the stemness factors BMI1, CD44, CD133, OLFM4 and c-MYC [45]. Their results demonstrated the double-negative feedback loop between miR-34a and Snail. In this study, we demonstrated that the inhibitory effect of miR-34a on tumor regeneration, metastasis, and clonogenic expansion was due to the downregulation of RTCB.

As of today, the knowledge regarding the function of this 3’-phosphate RNA ligase RTCB in disease progression is still limited. RTCB has been shown to catalyze tRNA splicing and involve in other RNA repair reactions by joining an RNA strand ending with a 2’,3’-cyclic phosphate to the 5’-OH group of another RNA strand in a GTP-dependent manner [46]. Furthermore, RTCB was implicated in the UPR as it was able to ligate the X-box binding protein 1 (XBP-1) mRNA during endoplasmic reticulum stress [35]. In this study, we showed that RTCB was a direct target of miR-34a using the luciferase reporter assay. Our results showed that the expression of RTCB was attenuated in the miR-34a-overexpressing HNC-TICs. HNC data from TCGA revealed that there was an inverse relationship between miR-34a and RTCB. Most importantly, the repressive effect of miR-34a on TICs properties was reverted by an increase in RTCB, which implied that RTCB mediated the self-renewal, invasion and colony-forming abilities in HNC-TICs. Furthermore, results from the in vivo experiment were in line with the in vitro findings. We showed that the administration of genistein retarded tumor growth and upregulated the expression of miR-34a in the excised tumors with downregulated expression of RTCB. Overall, these results indicated that genistein exerted its anti-tumor activities, at least in part, through the miR-34a/RTCB axis.

## 5. Conclusions

**Figure 8:**
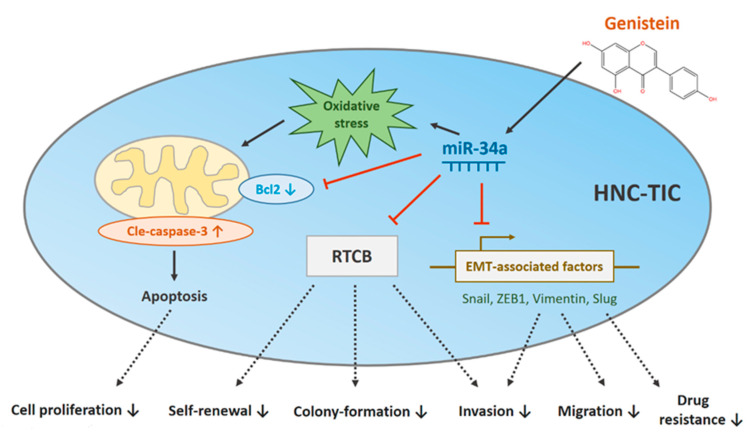
Proposed antineoplastic mechanism for oxidative stress-mediated genistein. HNC-TIC: tumor-initiating cells of head and neck cancers; ZEB1: zinc finger E-box binding homeobox 1; RTCB: RNA 2′,3′-cyclic phosphate and 5′-OH ligase.

In summary, our results demonstrated the antineoplastic mechanism of genistein (Figure 8). We showed that genistein inhibits the cell proliferation and aggressiveness of HNC-TICs via the upregulation of miR-34a. The downregulation of Bcl2 and induction of oxidative stress by genistein results in apoptosis of HNC-TICs. Besides, genistein exerts a synergistic effect with commonly used chemotherapy agents and increases chemosensitivity by suppression of various EMT factors, leading to reduced invasion, migration and drug resistance. The forced expression of miR-34a mitigates numerous stemness features through direct regulation of RTCB, which participated in the stemness characteristics of TICs. These results suggest that genistein hinders the progression of HNC via miR-34a/RTCB axis and may serve as a promising adjunct to the nutritional management of HNC patients receiving chemotherapy. Our results unraveled a novel function of RTCB and provided insight into the genistein-mediated tumor-suppressive potential.

## Figures and Tables

**Table 1:** List of primer sequences used for Real-Time qRT-PCR in this study.

Primer Name	Forward Primers	Reverse Primers
E-cadherin	ATTCTGATTCTGCTGCTCTTG	AGTCCTGGTCCTCTTCTCC
Vimentin	CAATGTTAAGATGGCCCTTG	GGGTATCAACCAGAGGGAGT
Snail	GCAGCTATTTCAGCCTCCTG	GTTCTGGGAGACACATCGGT
Slug	GTGATTATTTCCCCGTATCTCTAT	CAATGGCATGGGGGTCTGAAAG
ZEB1	AGCAGTGAAAGAGAAGGGAATGC	GGTCCTCTTCAGGTGCCTCAG
GAPDH	CTCATGACCACAGTCCATGC	TTCAGCTCTGGGATGACCTT

**Table 2:** List of primary antibodies used for Western Blot in this study.

Antibody	Species	Dilution Ratio	Company
Snail	mouse	1:1000	Cell signaling technology
ZEB1	rabbit	1:1000	Santa cruz biotechnology
Vimentin	mouse	1:1000	Santa cruz biotechnology
Slug	mouse	1:1000	Santa cruz biotechnology
E-cadherin	mouse	1:1000	Santa cruz biotechnology
RTCB	rabbit	1:1000	Thermo fisher scientific
GAPDH	mouse	1:5000	GeneTex
